# Evidence of Programmed Death-Ligand 1 Expression in a Highly Inflammatory Prostate: A Literature Review and Our Experience

**DOI:** 10.7759/cureus.67726

**Published:** 2024-08-25

**Authors:** Maria Koleva, Angelina Mollova-Kyosebekirova, Dorian Dikov

**Affiliations:** 1 Department of General and Clinical Pathology, Medical University of Plovdiv, Plovdiv, BGR; 2 Department of Pathology, Jossigny Hospital, Jossigny, FRA

**Keywords:** inflammation, pd-l1, prostate, lymphoepithelial lesions, prostatitis

## Abstract

Chronic inflammation (CI), a common finding in the human prostate, is associated with the most frequent socially important prostate diseases: prostatitis, benign prostatic hyperplasia, and prostate adenocarcinoma. Programmed cell death protein 1 (PD-1) and its ligand (PD-L1) expression are induced on the surface of immune and epithelial cells of healthy and tumor tissues in response to various cytokines. Here, we provide a comprehensive review of the PD-1/PD-L1 pathway in the non- and peri-tumoral inflammatory prostate, focusing on the structure and expression of PD-L1 and the diverse biological functions of PD-L1 signaling in health, high-grade CI (National Institutes of Health, category IV prostatitis or histologic prostatitis), and immune-related diseases, including autoimmunity, tumor microenvironmental immunity, and immune privilege. This review explores the possible pathophysiological interpretations of clearly visible, selective, and strong PD-L1 expression in the immuno-inflammatory-induced and related, histologically distinct sites of this expression: the ductal lymphoepithelial lesions and prostatic granulomas.

## Introduction and background

Chronic inflammation (CI) is frequently diagnosed in the human prostate. It is associated with prevalent and socially significant prostate diseases, including prostatitis, benign prostatic hyperplasia (BPH), and prostate adenocarcinoma (PCa). Extensive epidemiological, histopathological, and molecular pathological evidence underscores the pivotal role of inflammation in the development of these conditions [1,2]. In contrast to the urologist, the pathologist defines prostatitis as “more” inflammatory cells within the prostatic parenchyma [3]. The prostate has an important lymphoepithelial component responsible for genitourinary mucosal immunity, leading to the concept of prostate-associated lymphoid tissue (PALT) [4]. PALT shares immunological and microarchitectural features with secondary lymphoid organs like tonsils, particularly in terms of the lymphoid component. However, research on prostatic epithelial changes remains limited. Programmed cell death protein 1 (PD-1) and its ligand (PD-L1) expression are induced in both immune and epithelial cells in healthy and tumor tissues in response to various cytokines. The PD-L1 expression in benign prostatic tissue is a new field of research that can clarify the crucial role of CI in the development of socially important prostate diseases via a specific inflammatory and immunosuppressive microenvironment. PD-L1 expression is rare in primary PCa [5-7]. The marker is rarely investigated in the non-tumoral prostatic lesions. Limited data showed a PD-L1 expression associated with chronic indefinite inflammatory infiltrate [5-7]. Also, the contribution of the PD-1/PD-L1 axis in immune responses is poorly understood in prostatic CI. Investigating prostatic inflammatory histopathology for years, we conducted a study to quantitatively and qualitatively assess the PD-L1 (1:200, clone QR1, BIOCYC, Potsdam, Deutschland) immunohistochemical expression in 152 cases of various forms of CI of the prostate: National Institutes of Health, category IV prostatitis or histologic prostatitis (HP), various types of granulomatous prostatitis (GP), and the reactive lymphoid infiltrates near of BPH and PCa [8,9]. HP was classified as low or high grade (LG, HG) based on inflammatory severity [3,10]. Control groups included non-inflammatory autopsy prostates with no documented illness and prostates of newborns and prepubescent children [8,9].

## Review

PD-L1 expression in benign ductal lymphoepithelial lesions in HG-HP

**Figure 1:**
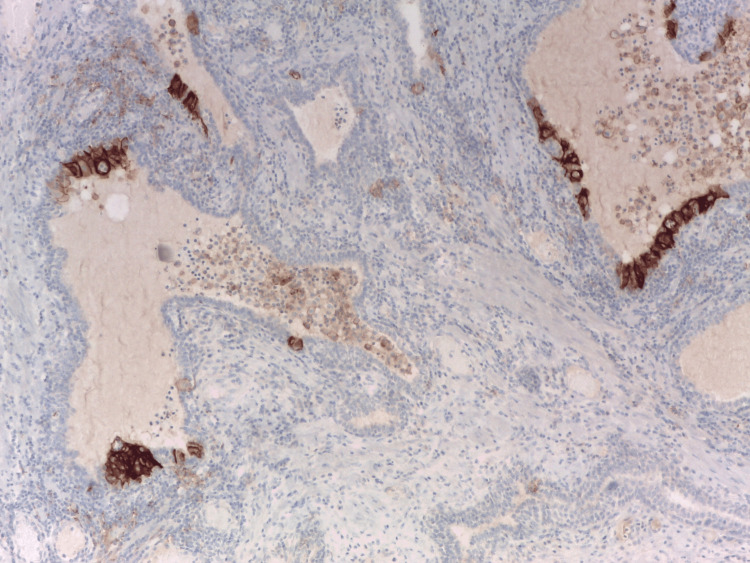
PD-L1 expression in HG-HP in serial sections. The epithelium of the lymphoepithelial lesion showed selective and strong membranous immunoexpression of programmed death-ligand 1, not needing a special indication with arrows. Immunohistochemistry anti-programmed death-ligand 1, x200. HG: high grade; HP: histologic prostatitis; PD-L1: programmed death-ligand 1 Image credits: Dr. Dorian Dikov

Strong and selective PD-L1 immunoreactivity was identified in the epithelium of morphologically distinct ductal structures, known as benign lymphoepithelial lesions (LEL), [8] within prostates affected by HP. Also, PD-L1 positivity in immune cells surrounding LEL, as well as in intraluminal macrophages (Figure 1).

**Figure 2:**
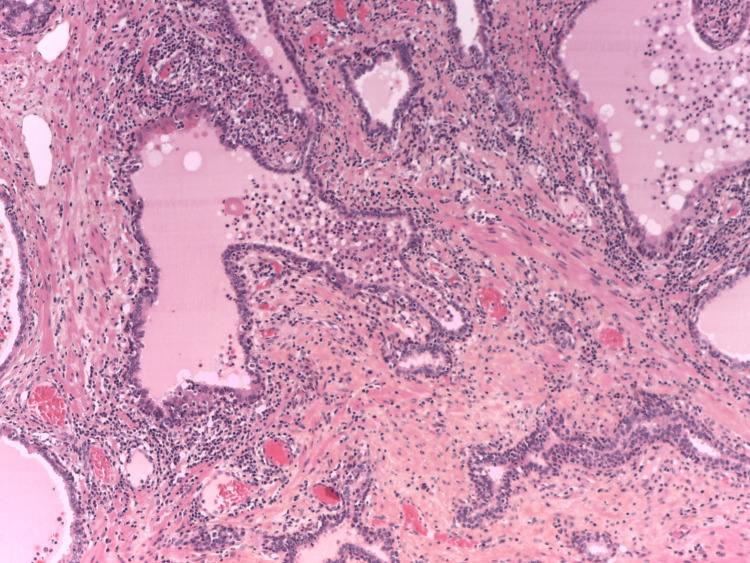
Chronic HG prostatic inflammation. Chronic high-grade (HG) prostatic inflammation with infiltration of lymphocytes affecting multiple prostatic ducts, associated with strongly eosinophilic epithelium, fully corresponding to programmed death-ligand 1-positive lymphoepithelial lesion from Figure 1; pronounced PD-L1 expression was also found in intra-luminal macrophages. Haematoxylin-eosin-saffron, x200. PD-L1: programmed death-ligand 1 Image credits: Dr. Dorian Dikov

Lymphoepithelial lesions (LEL) are distinct morphological changes in the ductal epithelium in the glandular organs. It is a histological finding seen in lymphoma of mucosa-associated lymphoid tissues (MALT) and benign inflammatory diseases with reactive or autoimmune pathogenesis [11-15]. LEL is defined as clusters of three or more lymphoid cells causing alteration of the epithelium, along with histological changes within epithelial cells, including pronounced eosinophilia (Figure 2) [11].

**Figure 3:**
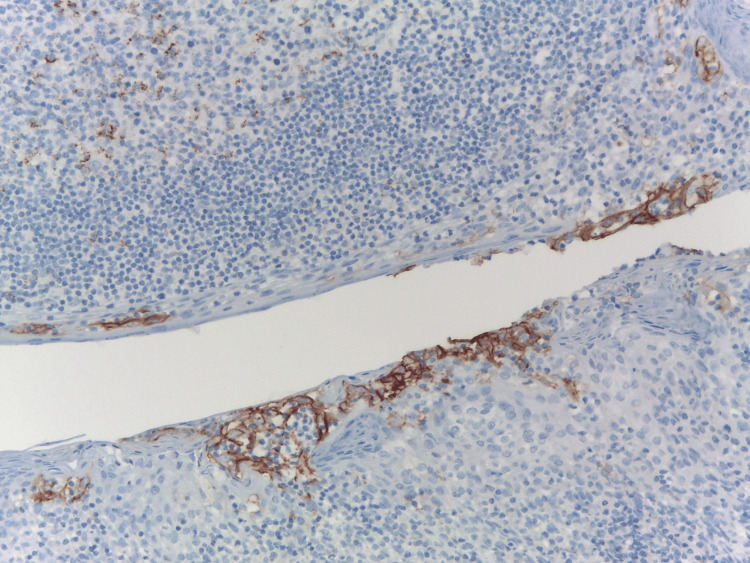
PD-L1 expression in tonsillar crypts lymphoepithelium of adenoids (external positive control). Immunohistochemistry anti-PD-L1, x200. PD-L1: programmed death-ligand 1 Image credits: Dr. Dorian Dikov

In malignant LEL found in marginal zone lymphomas, they are profuse, exhibit damage changes, and contain large, atypical centrocyte-like B-lymphoid cells [11]. In inflammatory diseases observed in other organs, benign LEL are small, non-destructive, and contain mature intra-epithelial lymphocytes (IEL) often with a dominance of T-cells [12,15]. We provide qualitative and quantitative analysis of LEL in the context of inflammatory human prostate conditions [8]. Prostatic LEL have no diagnostic organ specificity, even though they are a constant finding in HG-HP. Our statistical analysis revealed a strong correlation between the PD-L1-positive LEL and HG-HP (p<0.001, Fisher’s exact test) [8]. There was no significant PD-L1 expression or LEL in the control cases [8]. It is also discovered that intra-epithelial lymphocytes in both normal and inflamed prostates are primarily T-cells [16]. This peeks the interest in observation of PD-L1 in LEL. Expression of PD-L1 was also confirmed in the ductal epithelium of LEL in patients with CI, but not in normal or non-inflammatory prostate. This PD-L1 expression pattern correlates with the degree of lymphocytic inflammatory infiltration, resembling patterns seen in certain classical autoimmune diseases like Sjögren's syndrome [17]. The immune checkpoints, which are T-cell inhibitory molecules, prevent the activation of organ-specific antigen-reactive T-cells. To prevent tissue damage during inflammation, PD-L1 is normally overexpressed [18]. Unusual positivity for PD-L1 has been detected in various inflammatory conditions, including Helicobacter pylori+ chronic gastritis, bowel disease, salivary gland Sjögren’s syndrome, and celiac disease [13,19]. Prostatic ductal LELs are PD-L1-positive like these in autoimmune pancreatitis, chronic inflammatory diseases, and tonsillar crypts lymphoepithelium of adenoids (Waldeyer’s ring), the best external positive control in daily pathology practice (Figure 3).

Our speculation revolves around the notion that PD-L1 expression in LEL represents an immune response designed to curb persistent T-cell activation, thus preventing tissue damage and autoimmune T-cell-mediated prostatitis. This “adaptive or innate immune resistance” mechanism (immune suppressive microenvironment) may provide insights into the scarcity of intra-tumoral lymphocytic infiltration and PD-L1 expression in PCa, always with CI [20]. Our findings also highlight that LEL formation occurs in adult life and forms an integral part of the PALT and of the inflammatory prostatic microenvironment [8].

PD-L1 expression in granulomatous prostatitis 

**Figure 4:**
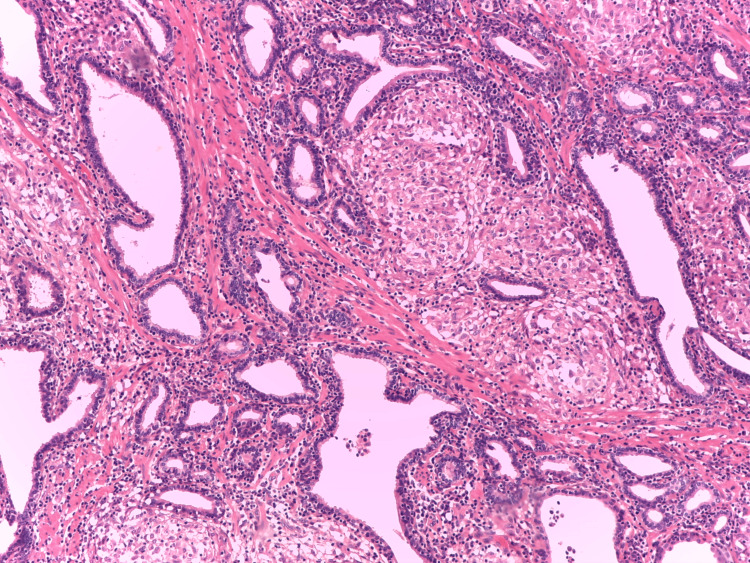
Foci of localized GP, haematoxylin-eosin-saffron, x100. GP: granulomatous prostatitis Image credits: Dr. Dorian Dikov

**Figure 5:**
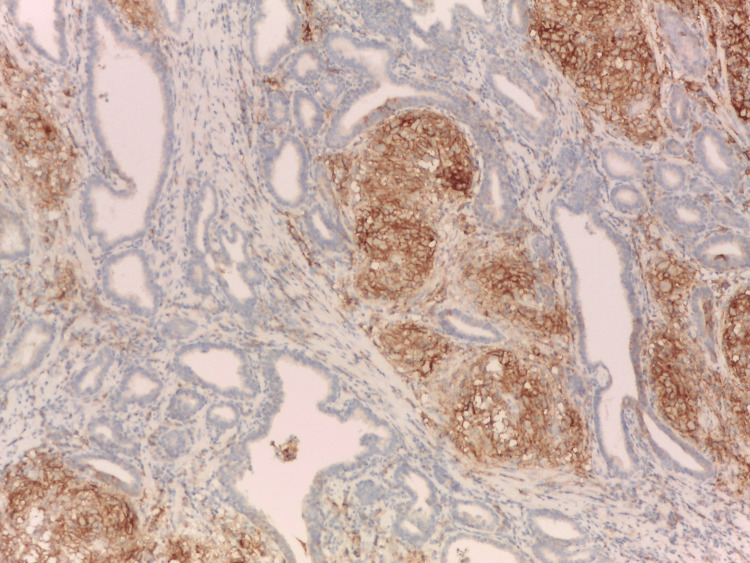
PD-L1 expression in GP in serial sections. Strong programmed death-ligand 1 (PD-L1) expression in the foci of localized granulomatous prostatitis. GP: granulomatous prostatitis Image credits: Dr. Dorian Dikov

In our previous study, a notable finding was the elevated PD-L1 expressions in GP [9], which contributes to enriching the profile of GP. PD-L1 expression is strong and membranous in both localized and diffuse granulomatous prostatic inflammation (Figure 4, 5) [9].

In all etiological forms of GP (HG by definition), the expression of PD-L1 is selective within the areas of macrophageal/epithelioid granulomatous inflammation. In GP, the PD-L1 staining was generally easy to interpret, with a combined positive score (CPS) between 75 and 97 [9]. GP manifests as a self-limited inflammatory process, typically presenting with clinical symptoms such as low-grade fever, dysuria, and frequency [21,22].Microscopically, this is characterized by diffuse lobular chronic inflammation with infiltration of macrophages, epitheliod macrophages, multinucleated giant cells, and granuloma formation [23]. The etiological classification of GP encompasses nonspecific granulomatous prostatitis (NSGP), infectious granulomas (Mycobacterial: Bacillus Calmette-Guérin (BCG), Mycobacterium tuberculosis, mycoses, and parasitoses), post-surgical granulomas, and systemic granulomatous prostatitis (sarcoidosis) [23]. GP is a microscopical diagnosis with particular attention given to differential diagnosis considerations, especially in distinguishing it from high Gleason grade PCa [24] and associated HG-HP [25]. The role that PD-L1 plays in the abnormally regulated T-helper immune responses observed in granulomatous inflammation is unclear. The selective expression of PD-L1 in epithelioid granulomatous lesions is a less-known fact in the daily practice of pathologists. Notably, Kubo T. et al., in the only published work, present the strong positivity of PD-L1 in some invasive granulomatous diseases, tuberculosis, sarcoidosis, Crohn’s disease, and foreign body granuloma [26]. Negativity in PD-L1 expression in our control cases (data not shown) and in other non-granulomatous macrophage-rich lesions shows that the staining is not a non-specific reaction and is related to the presence of PD-L1 protein in epithelioid histiocytes [9]. Epithelioid granulomas with multinucleated giant cells express PD-L1 provoked by IFNγ or TNFα. The same authors propose that these cytokines may play a common role in the formation of different types of granulomas [26]. From a general and immuno-pathological perspective, we can guess if the expression of PD-L1 in GP represents an immune response helping to prevent persistent T-cell activation, which could otherwise lead to severe tissue damage and autoimmune T-cell-mediated prostatitis [9].

PD-L1 as a diagnostic marker in HG-inflammatory prostate 

**Figure 6:**
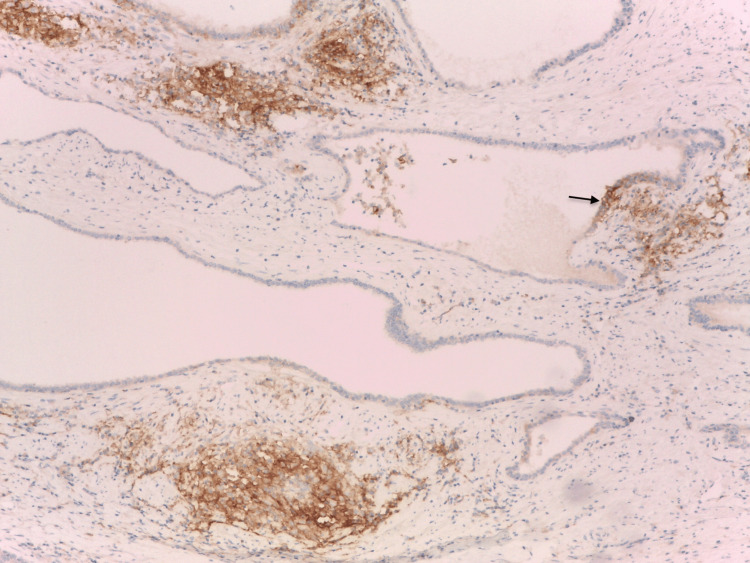
The strong and selective PD-L1 immunoreactivity in HG-inflammatory prostate is easily noticeable even under scanning microscopic magnification. The presence of four programmed death-ligand 1 (PD-L1)-positive stromal granulomas on the left side of the picture and one programmed death-ligand 1-positive lymphoepithelial lesion in the ductal epithelium in the right part (arrow). Immunohistochemistry anti-programmed death-ligand, x100. The strong and selective programmed death-ligand immunoreactivity in high-grade (HG) inflammatory prostate is easily noticeable even under scanning microscopic magnification: the presence of four programmed death-ligand-positive stromal granulomas on the left side of the picture and one programmed death-ligand-positive lymphoepithelial lesion in the ductal epithelium in the right part (arrow). Immunohistochemistry anti-programmed death-ligand 1, x100. Image credits: Dr. Dorian Dikov

Diagnosis of HP and GP relies on histological examination, with a primary focus on distinguishing them from high-grade Gleason PCa [24,25]. In this context, PD-L1 proves to be helpful for identifying prostatic epithelioid granulomatous lesions. All our positive cases (LEL in HG-HP and GP) showed clearly visible strong PD-L1 immunoreactivity, easily noticeable even under scanning microscopic magnification, and sometimes the two PD-L1-positive lesions are found in close proximity (Figure 6).

Except for LEL and GP-foci, the inflammatory cells in LG and HG-HP, BPH, tumor parenchyma, and stroma in PCa are negative for PD-L1 (data not shown) [9]. Prostatic ductal LEL are PD-L1-positive, like these in autoimmune pancreatitis of type 2 [27] and could probably serve as an immunomorphologic hallmark of the immunologic (autoimmune) phase of chronic prostatic inflammation.

Research Perspectives

**Figure 7:**
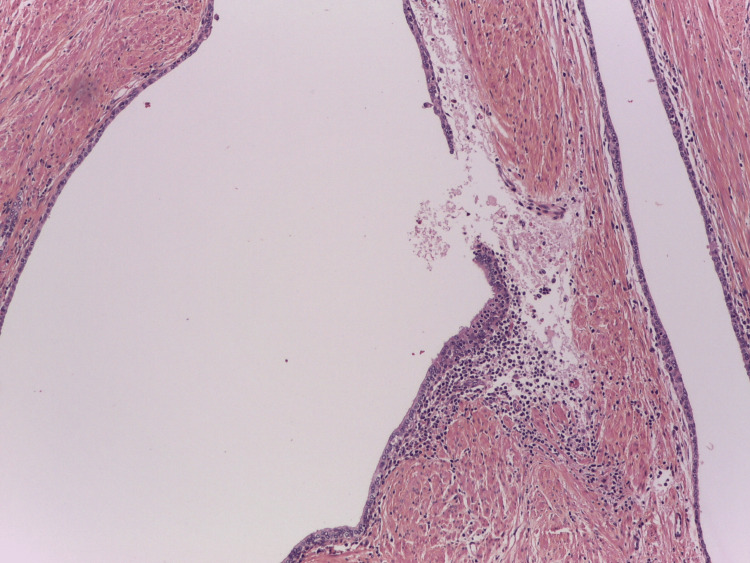
General view of HG-HP with rupture of a ductal LEL and diffusion of the intraluminal substances among immune periductal cells. Haematoxylin-eosin-saffron; x50. HG: high grade; HP: histologic prostatitis; LEL: lymphoepithelial lesions Image credits: Dr. Dorian Dikov

The PD-L1 expression in ductal LEL as a marker of prostatic epithelial barrier. Given that the prostate is an organ constantly exposed to pathogens, its ability to mount an early response to such stimuli is crucial for survival. However, the characteristics of the antigens that promote HP [2] and PD-L1-positive LEL formation are poorly understood so far [8]. In our previous study, we believe we are taking a step forward in clarifying the pathogenesis of HP, describing qualitatively and quantitatively the prostatic PD-L1-positive LEL [8]. The epithelium overlying the ductal LEL changes its functional morphology and immunohistochemical profile (PD-L1 expression), in order to become specialized for the transport of antigens directly from the ductal lumen. It is very possible that this specialized PD-L1-positive ductal epithelium plays a critical role as a gateway to the prostatic parenchyma. It has been shown to serve as an entry portal for antigens, like small intestinal and nasopharyngeal lymphoid tissues [28-30]. It is obvious that LEL are basic patho- and morphogenic elements of HG-HP, reflecting morphologically the moment of epithelial barrier disruption (Figure 7).

**Figure 8:**
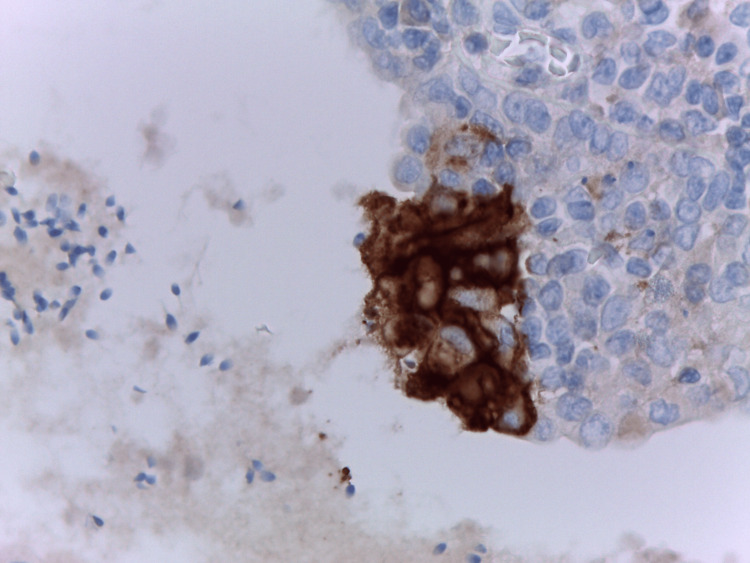
Damaged prostatic epithelium of ductal LEL in HG-HP. Programmed death-ligand 1-positive lymphoepithelial lesion in close proximity with intraprostatic spermatozoa (on the left). HG: high grade; HP: histologic prostatitis; LEL: lymphoepithelial lesions Image credits: Dr. Dorian Dikov

**Figure 9:**
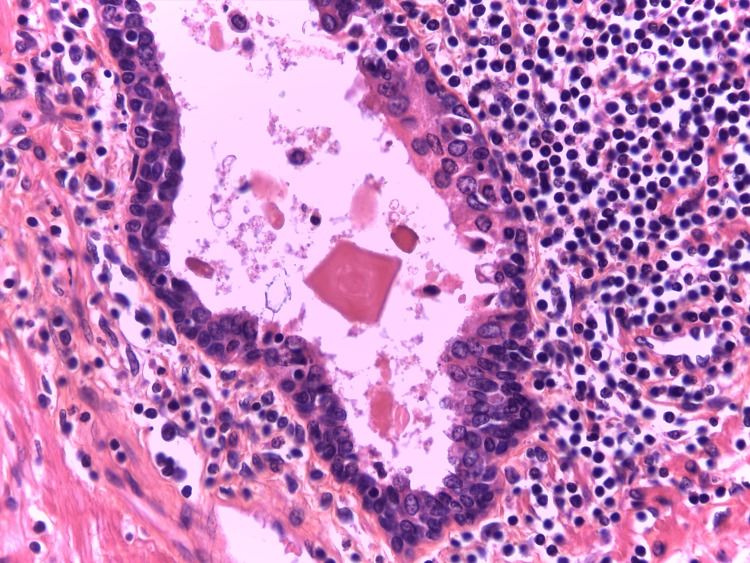
Eroded LEL from intraluminal corpora amylacea, haematoxylin-eosin-saffron, x400. LEL: lymphoepithelial lesions Image credits: Dr. Dorian Dikov

Obviously, this is followed by the influx of luminal antigenic substances that interact with the immune periductal cells (autoimmunization), leading to dysbiosis of the prostate microbiome and increased susceptibility to infection. Once initiated, this process can establish a feed-forward mechanism leading to a chronic, persistent HG inflammatory state (HG-HP) [2]. However, the specific initiator of the process, causing epithelial breakdown and inflammation, remains undefined. Prostatic epithelium could be damaged or disrupted for various reasons, including urine reflux and intraprostatic spermatozoa (Figure 8), and corpora amylacea (Figure 9).

The PD-L1 expression in HG-HP as a marker of immunosuppressive phenotype

**Figure 10:**
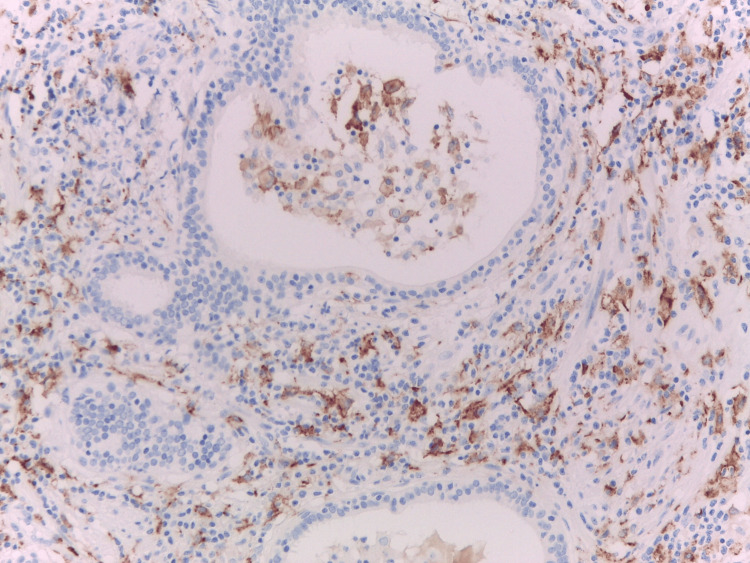
M2 intraprostatic macrophages in HG-HP, showing immunosuppressive phenotype CD163+. HG: high grade; HP: histologic prostatitis Image credits: Dr. Dorian Dikov

**Figure 11:**
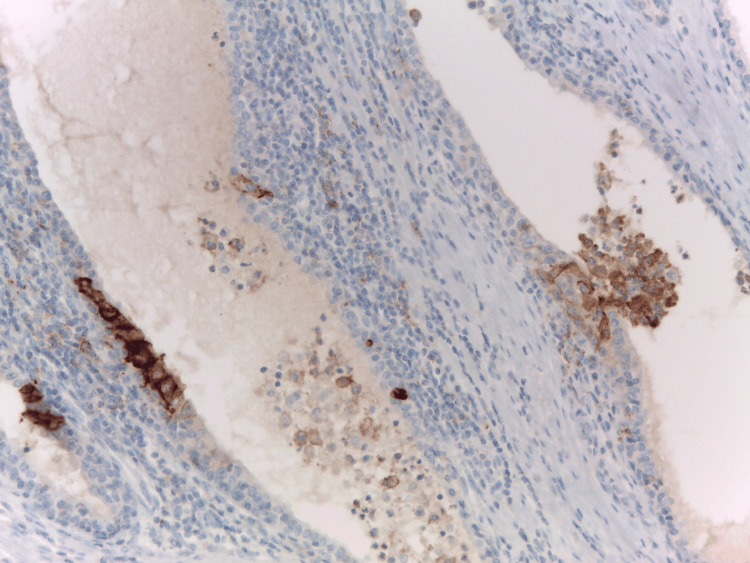
M2 intraprostatic macrophages in HG-HP, PD-L1+. HG: high grade; HP: histologic prostatitis; PD-L1: PD-L1: programmed death-ligand 1 Image credits: Dr. Dorian Dikov

The PD-1/PD-L1 axis constitutes an inhibitory pathway to moderate the mechanism of immune tolerance, providing immune homeostasis. The important immunosuppressive role of the PD-1/PD-L1 pathway in the tumor microenvironment and autoimmune diseases has been demonstrated [31]. In contrast to normal (inflammatory) macrophages (M1), tumor-associated macrophages (M2) favor local immunosuppression [32]. Along with CD163 expression (Figure 10), this immunosuppressive phenotype included PD-L1 positivity in M2 (Figure 11) [32].

The idea for the immunological independence of the prostate is based on factors such as the lack of afferent lymphatic vessels, a high incidence of occult carcinoma, the immunosuppressive properties of seminal plasma, and the normal low basal level of the intra-epithelial lymphocytes [16]. However, recent studies proved that the normal prostate is not an immunologically privileged organ; instead, it is a part of the mucosal immune system (MALT) [4,16]. In particular, the expression of PD-L1 in HG inflammatory prostate, both in the ductal epithelium of the LEL and in stromal and intra-glandular macrophages (M2), strongly suggests an immunosuppressive phenotype that developed in the course of CI [33]. This is a perfectly satisfactory explanation of this inflammatory-induced microenvironmental immune tolerance in the prostate. During inflammatory episodes in HG-HP or GP, when complete antigen elimination is inefficient, elevated PD-L1 expression remains high. The last is considered a sign of consumed T-cells [33]. Additional research is required to determine the potential role of immune checkpoint inhibitor treatments in the management of GP and HG-HP.

## Conclusions

In conclusion, our results and literature review provide evidence that PD-L1 is not expressed in normal human prostate and that expression of PD-L1 is induced by HG prostatic CI. PD-L1 positivity is located in a specialized, distinct ductal structure called LEL. LEL is not observed in the normal human prostate. PD-L1-positive LEL are an integral part of PALT and are inducible by external factors in terms of HG CI. PD-L1-positive LEL presents an integral part of HG-HP. HP (NIH, category IV prostatitis) occurs in several histologic patterns, and we describe a new pattern in prostatic inflammation, characterized by the presence of PD-L1-positive LEL in close association with HG-CI. Like other organs, PD-L1-positive LEL in the human prostate can be considered a hallmark of tissue autoimmunity and probably reflects the immune/autoimmune phase of HP (NIH category IV prostatitis). The association between PD-L1-positive LEL and HG-CI reveals the aggressiveness grade of the subclinical HP (NIH category IV prostatitis).

Our results could serve as an accurate guide for the clinician. We believe that except for the aggressiveness grades of the inflammation, the presence of PD-L1-positive LEL should be noted in the pathologist’s report, especially in initial prostate biopsies, in order to help the timing of the further biopsy. We suggest that the presence of PD-L1-positive LEL must be added to the actual NIH-consensus grading classification system based on morphological criteria for chronic prostatic inflammation. The novel data suggest that PD-L1-positive LEL may be a key player in the pathophysiology and morphogenesis of HP (NIH, category IV prostatitis) by mediating the self-sustained inflammatory immune/autoimmune processes in this chronic subclinical disease. In GP epithelioid granulomatous and diffuse inflammatory infiltrates exhibit selectively high levels of PD-L1 expression. In GP, the presence of PD-L1-positive LEL and granulomatous inflammation facilitates easy identification and diagnosis when stained with PD-L1.
